# Removing the barriers in health care services: the importance of emotional satisfaction

**Published:** 2018

**Authors:** Ș Spiridon, CM Gheorghe, IR Gheorghe, VL Purcărea

**Affiliations:** * Elias University Emergency Hospital, Bucharest, Romania; ** Department of Marketing and Medical Technology, “Carol Davila” University of Medicine and Pharmacy, Bucharest, Romania

**Keywords:** health care services, emotional satisfaction, WOM, loyalty

## Abstract

The competitive environment forces health care organizations to measure the quality and satisfaction as perceived by their health care consumers in order to determine both their financial and non-financial performance. The aim of this study was to examine the role of the consumers’ emotional satisfaction in health care services. More exactly, the study investigated the role of emotional satisfaction in two directions: as a consequence of perceived functional quality and as a precursor of the consumers’ behavioral intentions, such as loyalty and WOM, integrated into a conceptual model. The sample size was of 100 respondents, health care consumers of a private medical organization. The sampling method consisted of quota sampling, suggesting that each fifth individual received a questionnaire. The demographic profile of the health care consumers was determined in SPSS version 21 and the model was validated in SmartPls version 3, using the Structural Equation Modeling. Most of the respondents (51%) were male, from urban residential area (56%) and were aged between 28 and 37 years (40%). In terms of education, the vast majority had university degrees (47%), with their revenue between 2600-3000 RON (27%), married (51%) and who visited the doctor because of routine check-ups (47%). Moreover, the empirical model revealed that there are positive relationships between relationship quality and perceived quality, relationship quality and emotional satisfaction, perceived quality and loyalty, emotional satisfaction and loyalty as well as emotional quality and WOM.

## Introduction

The health care field has to overpass and adapt to the environmental pressures coming from the demographic changes in populations, the emergence of new treatments and technologies, as well as the high expectations of the internet consumers, the e-patients [**1**]. It is no surprise that many health care organizations develop strategies focused on service satisfaction and the behavioral consumer consequences such as loyalty and word-of-mouth (WOM).

Basically, health care services are different from other services if the consumer behavior is taken into consideration. Competitiveness among health care organizations depends on consumer specific constructs. As such, health care providers need to understand what is valued by health care consumers, how the quality and satisfaction of health care delivery are perceived, as well as the consumer behavioral consequences, materialized in how and when services should be improved.

The competitive environment forces health care organizations to measure the quality and satisfaction as perceived by their health care consumers in order to determine both their financial and non-financial performance. The advantages brought by the non-financial performance to health care organizations are improved service retention, positive WOM, reduced staff migration, decreased operating costs, and enlarged market share [**2**].

The concept of satisfaction has been a topic of great interest and, today, there are still some experts, who debate its importance and impact in practice. Simply, satisfaction is an outcome of the difference between what consumers expect and what they are delivered. Moreover, satisfaction refers to an insider’s perspective and perceived value [**3**]. However, in the health care context, consumers assess the cognitive and emotional outcomes across different time-points interactions with a health care organization. The quality perception, as well as satisfaction are influenced by certain characteristics of health care consumers such as the fact that they seldom determine their own needs and their decisions are taken strictly on subjective grounds, have limited ability to evaluate the outcome, and, of course, have no knowledge of the service attributes [**4**]. Despite the fact that there is entropy of information, health care consumers make decisions based on other service attributes, known in literature as functional quality [**5**]. Functional quality includes the caregivers’ thoroughness, experience, and communication skills [**6**].

Despite numerous studies attesting the importance of service quality and satisfaction in the health care setting, there has been relatively limited investigation on the determinants and consequences of emotional satisfaction. Further, there is a lack of empirical examination of the importance of emotional satisfaction in health care and specifically that choices are conducted on emotional grounds in health care services.

The aim of this study was to examine the role of the consumers’ emotional satisfaction in health care services. More exactly, the study investigated the role of emotional satisfaction in two directions: as a consequence of perceived functional quality and as a precursor of the consumers’ behavioral intentions such as loyalty and WOM.

A conceptual model was developed and empirically validated in the health care context. The proposed model was made up of the following constructs: relationship quality, perceived quality, emotional satisfaction, loyalty and WOM. Moreover, emotional satisfaction mediates the impact of perceived quality and the behavioral intentions such as loyalty and WOM [**7**].

## Literature review

**Emotional satisfaction**

Satisfaction has been depicted in scientific literature as a state [**8**]. This state of a person evolves into a fulfillment stage when it is integrated into a framework and it consists of 2 dimensions, reinforcement and arousal [**8**]. As such, in the case of low arousal, satisfaction becomes a contentment state and if the level of arousal is high, satisfaction evolves into a surprise, in positive and negative directions (delight versus shock), whereas if positive reinforcement occurs then satisfaction becomes pleasure and vice versa, if reinforcement has a negative evolution, satisfaction becomes relief [**8**].

Despite the vast amount of literature on the subject, there is a lack of consensus on whether satisfaction should be approached only by cognitive measurement scales, by emotional scales, or by a mixture between the two perspectives [**9**-**11**].

Taking into consideration the sensitive nature of the health care field, we believe satisfaction should be entirely an emotional construct [**1**][**11**]. Moreover, emotional satisfaction should not lack inconsistency when being measured, as consumption emotions are explored as discrete emotions or classified in general dimensions with negative and positive meanings [**12**].

In the health care context, satisfaction is an attitude shaped by an emotion, which should be measured by the total subjective assessments of multidimensional attributes associated with the health care experience [**13**]. Consequently, emotional satisfaction may describe the health care consumers’ evaluations of different experiences as moderated by the personal feelings of equity in the exchange process, the cognitive dissonance process that is the result between the desires and outcomes, as well as individual preferences and social comparisons [**14**]. Further, along with the upsurge of technology and especially the internet, many health care consumers express their satisfaction or dissatisfaction on Facebook groups or dedicated forums. Still, the most commonly encountered emotions related to the health experience are anger and rage, as they actually describe the emotional dissatisfaction [**15**].

**Perceived service quality**

According to Gronroos, the perceived service quality of a certain service may be described as “the result of an evaluation process, [in which] the consumer compares his expectations with his perception of the service received; in other words, he places the perceived service and the expected service opposite one another” [**16**]. Moreover, quality is considered a critical determinant of organization competitiveness and long-term profitability despite being a complex and confusing construct [**17**].

As described in the Marketing literature, the service quality construct focuses on the perceived quality and has been defined as a consumer’s judgement about an entity’s overall excellence and superiority [**18**].

In health care services, the Institute of Medicine reached a consensus in describing the perceived health care quality as the degree to which health services for individuals and populations increase the likelihood of desired health outcomes and are consistent with current professional knowledge [**19**].

What is acknowledged is that service quality is a construct made out of multiple dimensions [**20**][**21**]. According to Gronroos (1984), service quality should be split into technical quality (what is done) and functional quality (how it is done). Consumers perceive what they receive in the delivery process as the outcome of the process in which resources are allocated, suggesting in other words, the technical or the outcome quality of the process whereas how the process itself functions expresses the functional or the process quality dimension.

In some services, such as health care, the technical quality is difficult to evaluate as consumers lack the ability to evaluate the treatments prescribed as well as to understand the specific knowledge. However, they rely heavily on other measures of quality attributes associated with the process of health delivery such as reliability and empathy [**22**]. Further, Lim and Tang (2000) argue that consumers select a hospital based on the functional quality [**23**].

**Relationship quality**

Not until recently, has relationship quality been focused on and enabled specialists to agree that it exists and may have a vast array of outcomes [**24**].

From a marketing perspective, relationship quality refers to the overall depth and climate of a relationship [**25**]. Further, relationship quality explains the degree of accuracy of the established relationship between two entities and its closeness to expectations, predictions, goals, and desires [**26**].

According to Bateson and Hoffman (1999), during a service experience, various types of emotions may arise and further determine the behavioral consequences such as loyalty and WOM [**27**].

**Behavioral intentions**

Behavioral intentions are signals of whether consumers will remain with or defect from an organization and may be categorized as favorable or unfavorable [**28**]. The most favorable behavioral intentions refer to positive WOM and remaining loyal whereas unfavorable behavioral intentions include negative WOM and, as last option, take legal action against the organization [**29**]. a. Loyalty Loyalty is represented by a deeply commitment in the shape of a repeated buying behavior of a consumer [**30**]. Loyalty is also expressed as a preferred option in case of a needed action. b. WOM Today’s consumers believe more in the knowledge and options of other consumers because they already experienced the service. In the Marketing literature, WOM has been defined in various ways but, put into simple words, it is a form of communication, which is transmitted by sources that are assumed to be independent of an organizational influence [**31**]. Further, according to Westbrook (1987), WOM is an informal communication directed to other consumers of a certain service and the organization that sells it [**32**]. Positive WOM reflects the characteristics of the interpersonal communication among consumers in the shape of recommendations to other individuals or interpersonal discussions about pleasant, vivid, and new experiences [**33**]. WOM communication is more helpful and relevant in the service industries as it may reduce the perceived risk of the service delivery [**34**]. In the health care context, consumers engage in WOM communications with the scope of gaining more insight into a matter that would reduce their perceived risk and offer a chance to understand the service per se before the delivery and consumption [**35**]. Further, health care services being high in credence qualities, are assumed the suitable candidates for WOM communication among consumers [**36**].

**Conceptual framework**

**Fig. 1:**
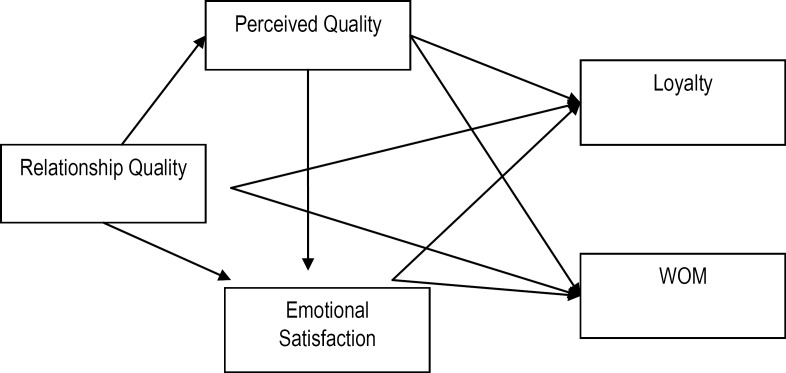
The proposed research model

Based on the preceding literature, a research model has been elaborated (**Fig. 1**). Along with the researched model, some hypotheses were proposed in order to be validated as they focused on the following relationships:

H1. Relationship quality is positively related to perceived quality.

H2. Relationship quality is positively related to emotional satisfaction.

H3. Relationship quality is positively related to loyalty.

H4. Relationship quality is positively related to WOM.

H5. Perceived service quality is positively related to emotional satisfaction.

H6. Perceived service quality is positively related to loyalty.

H7. Perceived service quality is positively related to WOM.

H8. Emotional satisfaction is positively related to loyalty.

H9. Emotional satisfaction is positively related to WOM.

**Materials and methods**

Based on the main findings of the literature review, in order to investigate the relationships among the constructs of the model, a self-administered questionnaire was elaborated and included different scales for every factor. As such, based on the explanation about functional quality as provided by Gronroos, the perceived service quality was assessed by items referring to the overall quality and delivery [**37**]. Emotional satisfaction was measured with items adapted from the works of Reynolds and Beatty (1999) as well as Wong (2004) [**38**][**39**]. As such, emotional satisfaction was captured with the help of a differential scale in which consumers would indicate their feelings regarding the health care service. The constructs Loyalty and Relationship quality have been measured using the scales proposed by Wong (2004) but adapted to the health care context [**39**]. WOM scale was assessed by using the items proposed by Gheorghe CM et al. [**22**].

The sample size was of 100 respondents, health care consumers of a private medical organization. The sampling method consisted of quota sampling, suggesting that every fifth individual received a questionnaire. The demographic profile of the health care consumers was determined in SPSS version 21 and the model was validated in SmartPls version 3, using Structural Equation Modeling.

## Results

**The demographic profile of the respondents**

The characteristics of the respondents are summarized in **Table 1**. A majority of the respondents (51%) were male, from urban residential area (56%) and with the age in the 28-37 interval (40%). In terms of education, the vast majority had university degrees (47%), with their revenue between 2600-3000 RON (27%), married (51%) and who visited the doctor because of routine check-ups (47%).

**Table 1:** The demographic profile of the respondents

Variables	Frequency	Percentage of total
Gender		
Male	51	51
Female	49	49
Residential area		
Urban	56	56
Rural	44	44
Age		
18-27 years	6	6
28-37 years	40	40
38-47 years	28	28
48-57 years	20	20
Above 58	6	6
Education		
Primary school	6	6
High school	26	26
University	47	47
Post university studies	21	21
Revenue (RON)		
Below 1200	5	5
1201-1599	7	7
1600-2000	25	25
2100-2599	22	22
2600-3000	27	27
Above 3000	14	14
Marital status				
Unmarried	44	44
Married	51	51
Widow	3	3
Don’t state	2	2
Type of medical consultation		
Routine consultation	47	47
Pre surgery consultation	21	21
Post surgery consultation	12	12
Second opinion consultation	20	20

**Descriptive statistics**

Means and standard deviations for all measures are reported in **Table 2**. All scales ranged from one to five. All items received mean scores between 3 and 3. 87, showing a rather neutral opinion.

**Table 2:** Descriptive statistics

Constructs and items	Mean	Standard deviation	Min	Max
Relationship quality			
It 1	3.80	1.41	1	5
It 2	3.87	1.30	1	5
Perceived quality			
It1	3.44	1.29	1	5
It2	3.46	1.23	1	5
It3	3.48	1.32	1	5
Emotional satisfaction			
It 1	3.79	1.32	1	5
It 2	3.65	1.25	1	5
It 3	3.62	1.15	1	5
Loyalty			
It 1	3.46	1.42	1	5
It 2	3.45	1.40	1	5
It 3	3.52	1.40	1	5
WOM			
It 1	3.51	1.30	1	5
It 2	3.65	1.26	1	5

**Reliability analysis**

Reliability checks were conducted on the multi-item measures using the Cronbach’s alpha coefficients as well as Composite Reliability. Moreover, it was also used the Average Variance Extracted for greater internal validity. All these values were assessed in SmartPls. According to Hair et al (2014) [**40**], Cronbach’s alpha coefficients and value of Composite Reliability should be greater than 0.7 whereas the values of Average Variance Extracted should be higher than 0.5. All the values illustrated in **table 3** indicate adequate reliability.

**Table 3:** The reliability values of the scales

	Cronbach’s Alpha	Composite Reliability	Average Variance Extracted (AVE)
**Emotional satisfaction**	**0,860**	**0,915**	**0,781**
**Loyalty**	**0,932**	**0,957**	**0,881**
**Perceived Quality**	**0,904**	**0,940**	**0,839**
**Relationship quality**	**0,827**	**0,920**	**0,852**
**WOM**	**0,899**	**0,952**	**0,908**

**Path analysis and hypothesis testing**

**Fig. 2:**
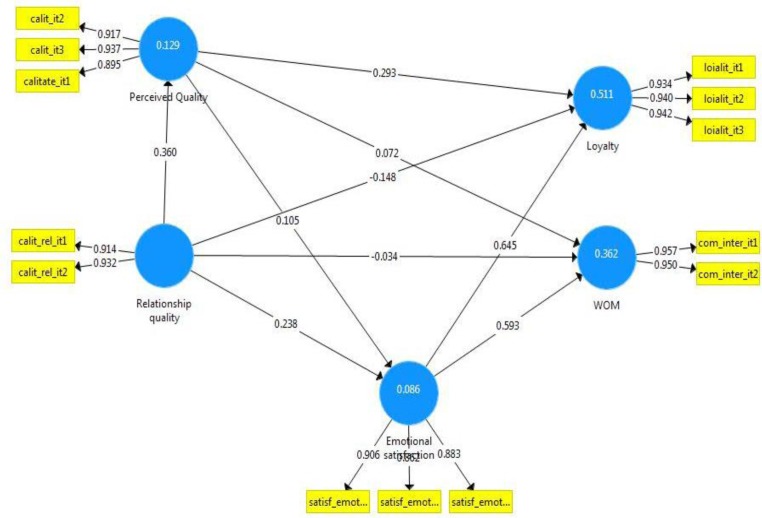
The results of the proposed model

As shown in **Fig. 2**, the path analysis for paths among the 5 major constructs were as follows: 1. The path leading from relationship quality to perceived quality had a coefficient of 0.360, with a p value of 0.001. The path was significant, which supports H1. 2. The path leading from relationship quality to loyalty had a coefficient of -0.148, with a p value greater than 0.05. The path was not significant, which does not support H3. 3. The path leading from relationship quality to WOM had a coefficient of -0.034, with a p value greater than 0.05. The path was not significant, which does not support H4. 4. The path leading from relationship quality to emotional satisfaction had a coefficient of 0.238, with a p value of 0.03. The path was significant, which supports H2. 5. The path leading from perceived service quality to emotional satisfaction had a coefficient of 0.105, with a p value greater than 0.05. The path was not significant, which does not support H5. 6. The path leading from perceived service quality to loyalty had a coefficient of 0.293, with a p value of 0.001. The path was significant, which supports H6. 7. The path leading from perceived service quality to WOM had a coefficient of 0.072, with a p value greater than 0.05. The path was not significant, which does not support H7. 8. The path leading from emotional satisfaction to loyalty had a coefficient of 0.645, with a p value of 0.001. The path was significant, which supports H8. 9. The path leading from emotional satisfaction to WOM had a coefficient of 0.593, with a p value of 0.001. The path was significant, which supports H9.

**Fig. 3:**
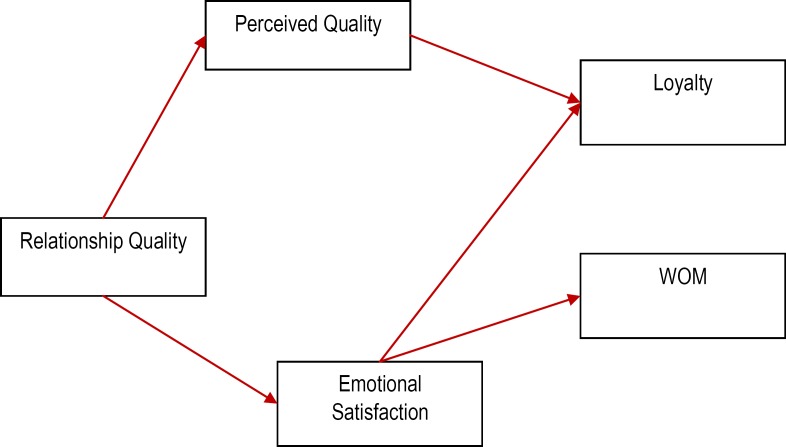
The refined model

Moreover, the refined model is depicted in **fig. 3**. The relationship quality explains 12% of the variance of the perceived quality construct as well as it explains 8% of the variance of emotional satisfaction. Perceived quality and emotional satisfaction explain 51% of the variance of loyalty and emotional satisfaction explains 36% of the variance of WOM.

## Discussion

The main objective of this study was to propose and test a model of the relationships among the constructs of relationship quality, perceived service quality and emotional satisfaction and behavioral intentions, via loyalty and WOM in the context of health care services. The conceptual model measured perceived quality only in terms of functional quality and relationship quality was postulated to have positive impacts on all other constructs included. Emotional satisfaction was posited to have direct relationships with the behavioral intentions, relationship quality, and perceived quality. Despite the fact that many case studies confirmed the antecedent role of perceived quality for emotional satisfaction, in this case study there was not confirmed any relationship. Similarly, perceived service quality was assumed to have a positive impact on WOM, but in this research, no significant impact was confirmed. This conclusion is not far from the results obtained in previous studies as they suggest an indirect effect is more plausible through satisfaction [**41**]. In this research, emotional satisfaction is influenced by relationship quality and not perceived quality.

The findings of the present study provide empirical evidence that in health care services, emotional satisfaction has an important role in defining service experiences. Several ideas may be withdrawn from this research, as follows: 1. Emotional satisfaction is derived from the service quality evaluation but the relationship side. A pleasant experience may encourage health care consumers to build a more lasting relationship with their physicians. These emotions tend mirror the consumer’s perceptions of how well the whole relationship fulfils their expectations, predictions and desires. 2. Emotional satisfaction has a positive impact on behavioral intentions. According to Yu and Dean (2001), emotional satisfaction was an important predictor of loyalty [**42**]. Thus, our findings are not far from other results reported in the scientific literature. 3. Emotional satisfaction had a positive impact on WOM. As such, the model confirmed that the more satisfied health care consumers are with their service delivery, the more are willing to use positive WOM. This result is in accordance with prior research findings [**22**]. 4. It seems that emotional satisfaction has a more important impact on loyalty than WOM. In the context of health care services, people are ashamed to talk about their health, as they perceive it as sensitive and intimate issues, suggesting that they do not feel comfortable to talk about them.

Specifically, from a strategic approach, understanding the consumer’s emotional displays can help enhance the overall service delivery and may be an effective asset of competitive advantage.

## Conclusions

The study investigated the role of emotional satisfaction in two directions: as a consequence of perceived functional quality and as a precursor of the consumers’ behavioral intentions, such as loyalty and WOM, integrated into a conceptual model. The empirical model revealed that there are positive relationships between relationship quality and perceived quality, relationship quality and emotional satisfaction, perceived quality and loyalty, emotional satisfaction and loyalty as well as emotional quality and WOM.
